# Assessing emergency room leadership of ObGyn residents in a public university teaching hospital of Sindh, Pakistan: a cross-sectional survey

**DOI:** 10.1186/s12909-024-05984-0

**Published:** 2024-09-13

**Authors:** Nusrat Shah, Nighat Shah, Umm-e Rabab, Hira Tariq, Samina Ayaz, Tanzila Fahim

**Affiliations:** 1 Vice- Chancellor-Shaheed Mohtarma Benazir Bhutto Medical University, Larkana, Pakistan; 2 Jinnah Postgraduate Medical Center, Karachi, Pakistan; 3 Present Address: Liaquat National Medical College, Karachi, Pakistan; 4 Department of Community Health Sciences, Aga Khan University, Karachi, Pakistan

**Keywords:** Decision making, Leadership, Karachi, Medical competencies, Medical residents

## Abstract

**Background:**

Leadership is a critical competency for medical professionals, yet it is often neglected in medical training. For ObGyn residents, leadership training is particularly crucial as it significantly impacts both maternal and newborn outcomes, as well as the operational efficiency of healthcare teams. The main objective of this study was to assess the perceptions of obstetrics and gynecology residents who served as group leaders in the emergency team at the Department of Gynecology, Ward 3, Dr. Ruth K.M. Pfau Civil Hospital Karachi.

**Methods:**

A Cross-sectional survey was conducted with purposively sampled 28 year-4 residents who worked as group leaders during last 3 years (from 2018 to 2020) of their residency program at the emergency team in the department of Gynecology Ward 3 Dr Ruth KM Pfau Civil Hospital Karachi. The perceptions on leadership were assessed on 25 items scale sent through a questionnaire on email. Grading of responses was done using a 4-point ordinal scale where 1 meant little importance and 4 was regarded as having great importance. Data was summarized with relevant descriptive statistics and was analyzed on SPSS version 22.

**Results:**

The mean age of residents was 30.36. The mean leadership scores of the group of residents were calculated to be 77.50 (SD ± 9.57) while 14(50%) residents showed good and 14 (50%) showed excellent leadership skills based on cumulative scores. Of the 25 traits examined in this study, the highest reported trait was humility 3.82 (± 0.39) followed by empowerment 3.68 (± 0.77) and effective communication 3.68 (± 0.77). While responding about learning experiences, 89.3% of participants felt that the experience enhanced their decision-making skills and boosted their confidence in dealing with emergencies.

**Conclusion:**

Our study highlights the critical importance of leadership development in the training of ObGyn residents, particularly in high-pressure emergency settings. The findings reveal that residents value leadership traits such as humility, empowerment, and effective communication, which are essential for building teamwork and ensuring optimal patient outcomes and patient satisfaction.

**Supplementary Information:**

The online version contains supplementary material available at 10.1186/s12909-024-05984-0.

## Introduction

Leadership can be defined as a process of influencing, motivating, and empowering other people to achieve a specific goal [1, 2]. Physicians, as collaborative leaders, can significantly improve healthcare outcomes, especially in emergencies where quick decision-making and efficient team management are vital. The CanMEDS defines the leadership competency for physicians as the ability of a physician to engage in shared decision-making for the operation and ongoing evolution of the health care system [3]. Pakistan Medical and Dental Council also highlights leadership as one of the 7 core competencies of a seven-star medical graduate of Pakistan [4]. However, although leadership is a required competency for undergraduate and postgraduate medical graduates, this competency is not explicitly taught or assessed in most medical education curricula [5].

Effective clinical leadership is the ability of a clinician to manage and lead a diverse set of team members to achieve good patient outcomes and reduce mortality and morbidity rates. Pakistan has currently one of the highest rates of maternal mortality in South Asia accounting for Maternal Mortality Rate (MMR) of 299 per 100,000 live births [6]. Traditionally the model of a clinical leader was an authoritarian leader who gave orders to team members. In contrast, modern clinical leadership is about teamwork and collaboration between immediate and extended team members to improve patient outcomes. Evidence shows that training of physicians in clinical leadership will lead to achieving better clinical outcomes. However, there are few systematic training programs which train the residents in clinical leadership skills [7].

Larsen et al. have suggested that since residents must manage emergencies in complex, chaotic and stressful conditions, they should be given the opportunity to practice leadership skills in real life emergency situations in order to develop courage and self-confidence as a team leader [8].

For ObGyn residents the leadership training is very crucial, as it significantly impacts both mother and newborn outcomes as well as the operational efficiency of healthcare teams. Obstetric and gynecologic emergencies often involve high-stakes situations where rapid decision-making, effective communication, and coordinated action are critical. For instance, conditions like postpartum hemorrhage, uterine rupture, eclampsia, or obstetric emergencies during childbirth require immediate and decisive intervention to prevent severe complications or fatalities for both mother and child [9, 10]. This was highlighted in qualitative study conducted in Pakistan with ObGyn residents and elaborated the hardships faced in terms of resources and teamwork while dealing with critical situations [9]. The role of leadership extends beyond managing clinical tasks; it also involves ensuring clear communication among team members, effectively delegating tasks, and fostering a collaborative environment [9].

Our postgraduate trainees (PGs) are the frontline physicians for managing critical obstetrical emergencies in tertiary care hospitals across the country. These junior doctors will form the bulk of next generation of expert physicians and will be expected to take up leadership roles in the healthcare system of the country. Hence it is important to develop their leadership and team building skills to enable them to run the system with efficiency [11].

Postgraduate residents (PGs) working in public sector hospitals in Pakistan manage emergencies in difficult and unpredictable conditions with limited facilities and human resource [10]. PGs working in obstetrics and gynecology of Civil Hospital Karachi (CHK) face a multitude of challenges. They manage critical patients referred from not just within Karachi and its outskirt, but also from remote areas from interior of Sindh and neighboring areas of Balochistan. In Gynae unit 3 of CHK, the protocol for group leadership (GL-ship) of emergency duties was changed in 2018. It was decided that every resident should get a chance to become the group leader (GL) for 3 months before they complete residency training. The GL should be the senior most year 4 resident and will be responsible for decision-making regarding patient management in emergency room (ER), labor room (LR), emergency operating rooms (EOTs) and wards, for delegating work among the team members, for reporting to on call consultants and also for solving the administrative issues. This study is being conducted to assess the perceptions of ex-GLs of Gynae unit 3 about their GL-ship training experience.

The rationale for this study is to know if the strategy of assigning every resident as a group leader of emergency team in year 4, is an effective teaching method for clinical leadership skills, so that this strategy can be further developed as a structured leadership training program and recommended for being incorporated in the obstetrics and gynecology (ObGyn) postgraduate training curriculum. The main objective of this study was to assess the perceptions of obstetrics and gynecology residents as group leaders of emergency team in department of Gynecology ward 3 Dr Ruth KM Pfau Civil Hospital Karachi.

## Methodology

A Cross-sectional survey was conducted for a period of one month after getting approval from IRB (from 15 January, 2022 to 15 February, 2022) Gynae unit 3, Dr Ruth K M Pfau, Civil Hospital Karachi. All the 28 year 4 residents who completed their 3-month Group Leadership (GL-Ship) for managing emergencies and who were relieved from the department after completing their residency training, from 1st January 2018 to 31st December 2020 were purposively sampled and invited to participate in the study. The sample size for this study was determined based on the total population of eligible participants, specifically all year-4 ObGyn residents who completed their residency and GL-ship for managing emergencies at the hospital. Year-4 residents are the only ones who are assigned leadership roles like Chief Resident and Team Lead, during their final year of residency. Therefore, all year 4 residents were included, ensuring that no eligible participant was left out. Year 4 residents are the ones who have completed there 3 years of FCPS training and are now in their final year of training. This approach led to a total of 28 residents being selected, representing a complete census of the eligible population. Purposive sampling was selected to specifically target year-4 residents who had served as group leaders (GLs) in emergency settings at a university teaching hospital. The rationale behind choosing this sampling method lies in the unique leadership experiences of these residents. Since they were deliberately chosen for their role as GLs, they possess specific insights into leadership in high-pressure emergency environments, which is the focus of this study.

A structured and anonymous self-administered questionnaire was used for data collection. IRB approval was taken from the ethical review board and informed consent was obtained from all residents. It was emailed to all residents who worked as GLs during the last 3 years from January 2018 to December 2020. The questionnaire had demographic data relating to age, year since completion of residency and marital status of the resident, 25 items related to leadership qualities and three open ended questions. Grading of responses was done using a 4-point ordinal scale where 1 = of little importance and 4 = of great importance. The main outcome measures were perceptions of ex-GLs about their learning experiences as GL of the emergency team.

## Development of the leadership assessment questionnaire

The 25-item leadership assessment questionnaire was adapted from the Leadership Practices Inventory (LPI) by Kouzes and Posner [12], and the Multifactor Leadership Questionnaire (MLQ) by Bass and Avolio [13]. The LPI evaluates leadership across five key practices: Model the Way, Inspire a Shared Vision, Challenge the Process, Enable Others to Act, and Encourage the Heart. The MLQ measures transformational and transactional leadership styles, including Idealized Influence, Inspirational Motivation, Intellectual Stimulation, Individualized Consideration, Contingent Reward, and Management-by-Exception.

**Table:** 

Adaptation from Leadership Practices Inventory (LPI)	Adaptation from Multifactor Leadership Questionnaire (MLQ)
Effective Communication: Enable Others to Act	Critical Thinking: Intellectual Stimulation
Visionary: Inspire a Shared Vision	Problem Solving: Intellectual Stimulation
Motivation: Encourage the Heart	Acuity: Idealized Influence
Integrity: Model the Way	Conflict Resolution: Individualized Consideration
Supportive: Enable Others to Act	Empowerment: Individualized Consideration
	Resilience: Inspirational Motivation
	Accountability: Contingent Reward
	Agility: Intellectual Stimulation
	Spread Positivity: Inspirational Motivation

To ensure the reliability and validity of the assessment tool used in our study, the leadership assessment questionnaire was adapted from established tools like the Leadership Practices Inventory (LPI) by Kouzes and Posner, and the Multifactor Leadership Questionnaire (MLQ) by Bass and Avolio. These tools are widely recognized in the field of leadership assessment and have been validated in various studies.

### Validation process

The adapted questionnaire underwent content validation by an expert in medical education, ensuring that the items were relevant and appropriately reflected the leadership skills intended to be measured in the context of ObGyn residency. The content validation process involved a thorough review by an expert in medical education for refinement of the questionnaire items to align them with the study's objectives. Special attention was given to the cultural and social context of the questions, ensuring that they were relevant and applicable to the local environment in which the residents were trained. This approach ensured the accuracy of capturing the residents' perceptions and experiences within their specific sociocultural setting.

### Pilot testing

Following this, the finalized questionnaire was pilot tested on a small sample comprising 10% of the residents from a different hospital who were not part of the main study. This pilot testing aimed to assess the clarity, comprehensibility, and overall functionality of the questionnaire. Feedback from the pilot test participants was used to make minor adjustments to the wording and format of the questionnaire to enhance its clarity and ease of use.
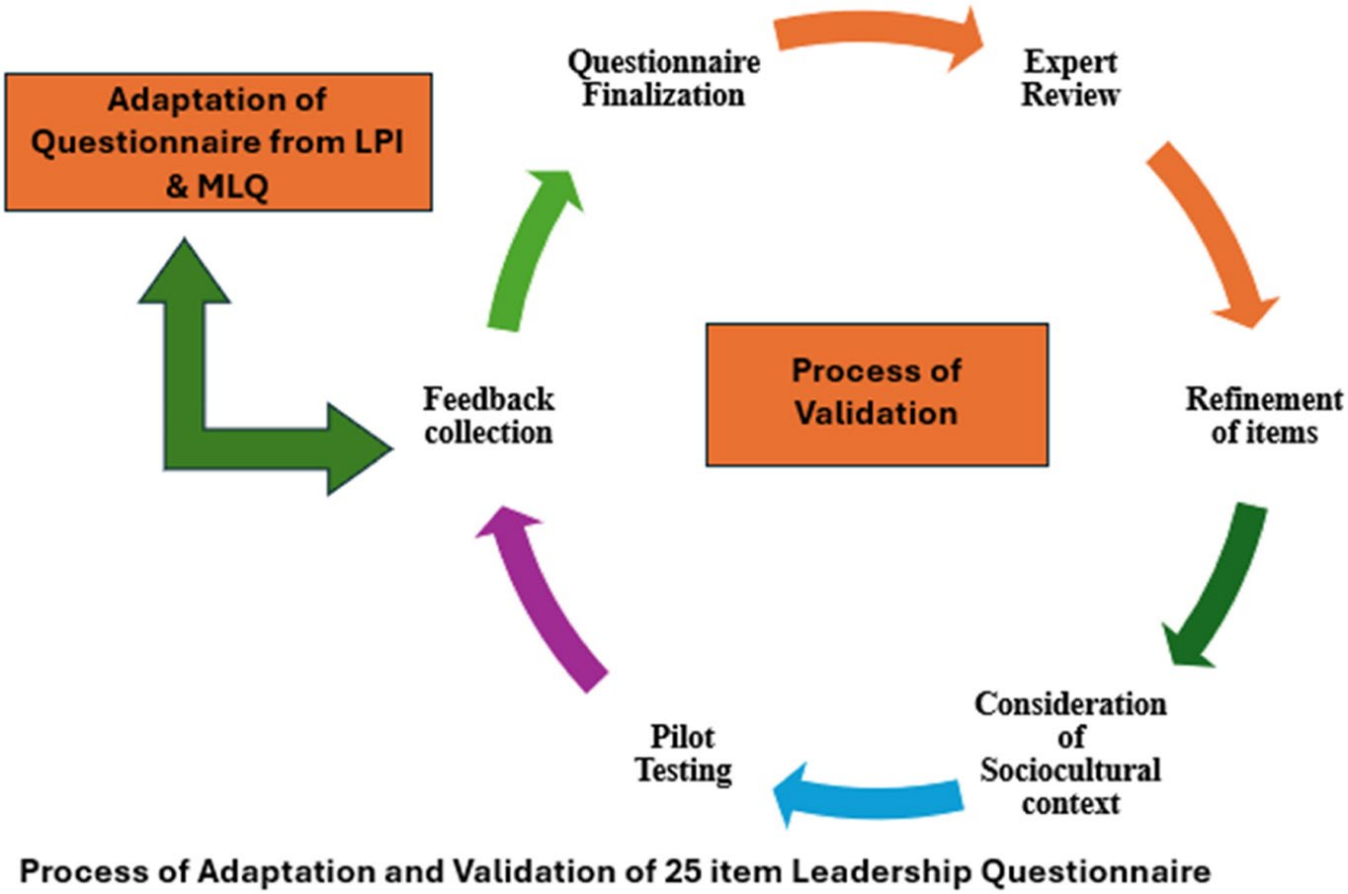


### Data analysis

Data entry and statistical analysis was done using statistical package of social sciences (SPSS) 22. SPSS version 22 has a user-friendly interface, which facilitates comprehensive data analysis. SPSS is well-supported in the academic community, which ensures the reproducibility and credibility of the results. Descriptive statistics were used to summarize the demographic characteristics of the study participants, including age, marital status, and years since completion of residency. Means, standard deviations, frequencies, and percentages were calculated to provide a clear overview of the data. The perception of leadership traits was also summarized using mean and standard deviation.

All responses were thoroughly checked for completeness. Given the small sample size (*n* = 28), missing data were minimal and were handled using listwise deletion, ensuring that only complete cases were included in the final analysis.

## Results

The mean age of residents was 30.36 (SD ± 1.42) years. Among them 57.1% (*n* = 16) were married while 42.9% (*n* = 12) were unmarried. The average time since residency was completed was 30.36 (SD ± 1.42) months (Table 1).

**Table 1:** Descriptive characteristics of participants (*n* = 28)

Variable	Mean (SD) & Frequency (%)
**Age(years)**	30.36 ± 1.42
**Years since completion of residency (months)**	30.36 ± 1.42
**Marital Status**	
Married	16 (57.1%)
Unmarried	12 (42%)

Key findings regarding leadership traits as well as overall trends in residents’ leadership development are shown in Table 2. The mean leadership scores of the group of residents were calculated to be 77.50 (SD ± 9.57) while 14(50%) residents showed good and 14 (50%) showed excellent leadership skills based on cumulative scores (Table 2).

**Table 2:** Leadership scores and skills of participants (*n* = 28)

**Variables**	Mean (SD) & Frequency (%)
**Leadership Scores**	77.50 ± 9.57
**Leadership Skills**	
Good	14 (50%)
Excellent	14 (50%)

Of the 25 traits examined in this study, on the level 1–4 scale, the highest reported trait was Humility/ Humble nature 3.82 (± 0.39) followed by Empowerment 3.68 (± 0.77) and effective communication 3.68 (± 0.77). Resident also considered Integrity and active listening 3.64 (± 0.62) as important traits for leadership (Table 3). The high rating of humility suggests that residents valued a leadership style that is collaborative and inclusive. In the high-pressure environment of an emergency room, a humble leader who listens to team members and acknowledges their input can create a more cohesive and effective team. Also empowerment indicates that residents see the importance of building autonomy and confidence in their team members where quick decision-making is crucial and becomes a matter of life and death. This reflects the residents' experiences of being entrusted with responsibilities. The high rating for effective communication highlights its critical role in emergency situations where miscommunication can lead to errors and adverse outcomes. Residents likely rated this trait highly because they understand that clear communication is essential for coordinating efforts, especially in a multidisciplinary team setting where the stakes are high.

**Table 3:** Summary of perceptions of residents on leadership traits (*n* = 28)

Leadership Traits	Mean (SD)	Great importance (%)	Marked importance (%)	Modest importance (%)	Little importance (%)
1.Critical Thinking	3.11 ± 1.06	46.4	32.1	7.1	14.3
2.Problem solving	3.21 ± 0.99	50	32.1	7.1	10.7
3.Learner	3.46 ± 0.79	60.7	28.6	7.1	3.6
4.Active listening	3.64 ± 0.62	71.4	21.4	7.1	–
5.Motivation	3.50 ± 0.83	64.3	28.6	–	7.1
6.Passionate	3.14 ± 1.20	57.1	21.4	–	21.4
7.Effective communication	3.68 ± 0.67	78.6	10.7	10.7	–
8.Acuity	3.57 ± 0.83	71.4	21.4	–	7.1
9.Divergent thinking	3.36 ± 0.82	53.6	32.1	10.7	3.6
10.Conflict Resolution	3.36 ± 0.82	53.6	32.1	10.7	3.6
11.Visionary	3.18 ± 0.94	42.9	42.9	3.6	10.7
12.Agility	3.00 ± 1.05	39.3	35.7	10.7	14.3
13.Integrity	3.64 ± 0.67	71.4	25.0	–	3.6
14.Accountability	3.50 ± 0.69	57.1	39.3	–	3.6
15.Respectful	3.46 ± 0.88	64.3	25.0	3.6	7.1
16.Humility/ Humble	3.82 ± 0.39	82.1	17.9	–	–
17.Streamlining resources	2.21 ± 1.06	14.3	25.0	28.6	32.1
18.Inquisitiveness	2.96 ± 0.92	28.6	50.0	10.7	10.7
19.Resilience	1.61 ± 0.95	7.1	10.7	17.9	64.3
20.Spread positivity	2.18 ± 1.15	17.9	21.4	21.4	39.3
21.Empathetic	3.00 ± 1.12	42.9	32.1	7.1	17.9
22.Mind Mapping	2.82 ± 1.33	46.4	21.4	–	32.1
23.Supportive	1.96 ± 1.20	17.9	14.3	14.3	53.6
24.Discipline	2.43 ± 1.10	14.3	46.4	7.1	32.1
25.Empowerment	3.68 ± 0.77	82.1	7.1	7.1	3.6

Some traits, such as Resilience (Mean = 1.61 (SD = 0.95) and Supportive (Mean = 1.96 (SD = 1.20), were rated lower in importance. The lower rating for resilience may suggest that the residents prioritize interpersonal and communication skills over personal coping mechanisms in their leadership roles. lower rating for being supportive might reflect the residents' focus on more active, outcome-oriented traits like communication and empowerment.

While responding about learning experiences during 3 months of leadership 89.3% of participants think that the experience enhanced their decision-making skills and boost their confidence to deal with emergencies in ER and OPDs independently. Among all participants 64.3% thought that they were able to deal with non-favorable situations and have handled stressful conditions perfectly. Almost 43% participants reported improved communication skills and the ability to interact with colleagues, staffs, patients and attendants.53.6% participants reported great experience about team management and team building.

The most common challenge reported was limited resources including less availability of beds, shortage of staff, difficulty to arrange operation theaters and surgical supplies. Next on the list was a conflict issue between doctors, staffs, paramedics and OT technicians (46.4%). Most of the residents (42.9%) reported non cooperation from staff/ OT technicians as a major concern to deal with. Arranging blood and blood products was also reported as a big concern by residents (25%) and a main limitation towards effective and timely management of critically ill patients in ER and OTs. Patient overload as compared to available resources was also a big challenge reported by many residents (21.4%) towards quality care. Administrative issues (17.9%) and dealing with difficult and violent attendants (17.9%) also created a big challenge for doctors. favoritism from seniors for some residents and lack of communication with others was reported by (17.9%) of residents. few participants also reported Language barrier as a challenge to treat patient effectively.

## Discussion

In this study, the group leaders were asked to review their leadership journey. Most of them cherished this period of their training as they manifested complex and comprehensive set of leadership skills. These are essential for effective teamwork, communication and patient safety as shown by other studies as well [14–16].

Unfortunately literature is not very abundant on building future medical leaders, but our study and clinical model put the residents in that position where they were uniquely placed to practice leadership in lifesaving situations on daily basis. Some authors have based their leadership programs on attributes such as confidence [17], communication skills [18], emotional intelligence [19] and organizational leadership [20]. There are no evidence-based, systematic proper outstanding programs however to cultivate a leadership culture, our study and model was an endeavor in that direction.

Our participants had a unanimous consensus that working as team leaders helped them make better teams, enhanced their leadership skills and provided a good impact on working environment. Existing leadership programs in graduate medical education, such as the one by Awad et al. [18], focus on broader communication skills. Awad et al. [18] implemented leadership training for surgical residents over 6 months. They aimed to improve collaborative leadership by fostering a communication style that is regarded less commanding. The authors were able to demonstrate significant increases in these areas; however, training effects in terms of leadership performance such as improved team interactions have not been evaluated.

Around a third of our participants were more receptive and adaptive to patient care and safety and a half of them expressed confidence in time and resource management. This has uptill now not seen in international studies. Our study hence goes beyond research and is not looking at a one time intervention as others [21] but is a model which is implemented and practiced with impactful results.

Evidence emphasizes that leadership skill set improves patient outcomes [22, 23]. The fragile healthcare system of Pakistan possess great challenges for the healthcare workers [10]. Our young leaders felt, it as a positive, productive and impressive learning journey. Some of them felt it as a great responsibility leading to confidence boosting and enhancing decision-making power. The challenges encountered by the residents during their GL-ship assignment, such as limited resources, staff shortages, and managing high patient loads, had a significant impact on the leadership qualities being tested through the questionnaire. These challenges directly influenced traits like decision-making, conflict resolution, and resource management. Scarcity of resources, a persistent challenge in tertiary care public sector hospitals, particularly tests the managerial characteristics of leaders as they must ensure the system runs smoothly despite these constraints. The four-year training program played a crucial role in helping residents meet these challenges, particularly in the Operation Theater (OR) and Emergency Room (ER) settings independently. This align with studies which have reported team management, conflict resolution, time management and dealing with administrative issues were major concerns faced by many residents which they managed with the help of senior doctors and reported it as a great learning venture [24–26]. Hence leading teams improved clinical, communication and managerial skills and had a positive impact on confidence level and patient dealing independently [9, 27].

The Accreditation Council for Graduate Medical Education (ACGME) core competencies and practice guidelines [28, 29], have suggested all those attributes that were learned during this phase including trust and responsibility towards patients and peers. The most reported suggestion was to be humble, polite and respectful to every member of the team as well as towards patients. Building good relations with team members is the key to effective teamwork and team coordination. Honesty with work, profession and patience can create a positive impact leading to the success of a doctor as a healer [30]. Staying focused, calm and target-oriented during difficult and stressful conditions is a great skill to learn and every resident should work on it [30]. Motivation and passion are the driving force towards any successful outcome and future Group leaders should work on it. In the last every resident had emphasized the importance of good communication skills and suggested every future group leader to improve communication with their colleagues, staffs and patients.

## Strengths and limitation

To our knowledge leadership in graduate medical education has not been evaluated widely in literature. Especially when it comes to quantifying the traits of leadership and assigning the level of importance that is given to each trait. This study looks at a continuous leadership implementation program, hence summarizing a three year and ongoing program to date and assessing residents perceptions on the strengths and weaknesses.

### Limitations

The study has some limitations and potential biases. The small sample size of 28 year-4 ObGyn residents and being a single center study conducted in public teaching hospital limits the generalizability of the findings. Conducting the study in a single center further limits the external validity, as the unique training environment at this hospital might differ from other institutions. Additionally, the reliance on self-reported data through questionnaires can introduce response bias or recall bias, where participants may have overstated or understated their leadership skills. The cross-sectional design of the study captures data at only one point in time, limiting the ability to assess changes in leadership skills or establish causality. Despite these limitations, the study provides valuable insights and highlights the need for future research to address these issues, such as including larger, more diverse samples and using longitudinal methods to better understand the development of leadership skills in ObGyn residents. Curriculum improvements with the integration of structured leadership modules into the residency program that focus on high-rated traits such as humility, empowerment, and effective communication. Real-life simulations of emergency scenarios can be used, allowing residents to practice and develop key leadership skills in a controlled environment. Additionally, mentorship programs where experienced clinicians guide residents through leadership challenges can provide valuable hands-on experience and feedback.

## Conclusion

Our study highlights the critical importance of leadership development in the training of ObGyn residents, particularly in high-pressure emergency settings. The findings reveal that residents value leadership traits such as humility, empowerment, and effective communication, which are essential for building teamwork and ensuring optimal patient outcomes and patient satisfaction. There is need for a more structured and systematic approach to leadership training within the ObGyn residency curriculum. By incorporating targeted leadership modules, real-life simulations, and mentorship opportunities, residency programs can better equip future clinicians with the skills needed to navigate complex clinical environments and lead healthcare teams effectively.

## Supplementary Information

Supplementary Material 1.

## Data Availability

The datasets used and/or analysed during the current study are available from the corresponding author on reasonable request.

## Abbreviations

CHK: Civil Hospital Karachi CHK
GL-Ship: Group Leadership
PG: Postgraduates
OPD: Out patient department
ER: Emergency Room
SPSS: Statistical Packages for the Social Sciences
ACGME: Accreditation council for graduate medical education

## Data availability

Data can be given on reasonable request to the corresponding author through email.
