# Supplementary material for: Assessing emergency room leadership of ObGyn residents in a public university teaching hospital of Sindh, Pakistan: a cross-sectional survey
Source: BMC Med Educ. 2024 Sep 13;24:1001. doi: 10.1186/s12909-024-05984-0 (PMC11401368; doi:10.1186/s12909-024-05984-0)
Supplement: Supplementary file 1 — Supplementary Material 1. [file 12909_2024_5984_MOESM1_ESM.docx]

| **Questionnaire** | | | | | | | | |
| --- | --- | --- | --- | --- | --- | --- | --- | --- |
| Sno. | Variable |  |  | | | | | |
| 1 | Age | ___________ |  |  |  |  |  |  |
| 2 | Marital Status | 1. Married 2. Unmarried |  |  |  |  |  |  |
| 3 | Years since completion of residency expressed in months | ____________ |  |  |  |  |  |  |
| Leadership Traits | | | | | | | | |
| Sno. | Traits | Question | Great Importance (%) | Marked Importance (%) | | Modest Importance (%) | | Little Importance (%) |
| 1 | Critical Thinking | I frequently question the usefulness of current practices |  |  |  | |  | |
| 2 | Problem solving | I can formulate solutions for difficult organizational issues |  |  |  | |  | |
| 3 | Learner | I develop in-depth knowledge of a range of clinical topics |  |  |  | |  | |
| 4 | Active listening | I listen to and incorporate my colleagues suggestions into decision-making |  |  |  | |  | |
| 5 | Motivation | I motivate the team members to have commitment to common goals |  |  |  | |  | |
| 6 | Passionate | I run rounds and make presentations effectively |  |  |  | |  | |
| 7 | Effective communication | I communicate with their own team members and inter-departmental team members effectively |  |  |  | |  | |
| 8 | Acuity | I make patients’ diagnostic and management decisions confidently |  |  |  | |  | |
| 9 | Divergent thinking | I listen to team members preferences and try to reach common ground |  |  |  | |  | |
| 10 | Conflict Resolution | I try to solve conflicts among team members before they become serious roadblocks |  |  |  | |  | |
| 11 | Visionary | I articulate a clear vision for how team members might work more closely together |  |  |  | |  | |
| 12 | Agility | I generate creative solutions in the midst of chaotic circumstances |  |  |  | |  | |
| 13 | Integrity | I show honesty and forthrightness in dealing with team members |  |  |  | |  | |
| 14 | Accountability | I show fairness and impartiality in dealing with team members |  |  |  | |  | |
| 15 | Respectful | I de1monstrate a caring attitude towards team members |  |  |  | |  | |
| 6 | Humility | I treat all team members equally irrespective of cultural, ethnic, and religious differences |  |  |  | |  | |
| 17 | Streamlining resources | I direct human resources to serve the team’s objectives |  |  |  | |  | |
| 18 | Inquisitiveness | I s1eek suggestions from team members in discussions which affect them |  |  |  | |  | |
| 19 | Resilience | I not only take risks but encourage team members to take risks |  |  |  | |  | |
| 20 | Spread positivity | I exhibit a sense of humor |  |  |  | |  | |
| 21 | Empathetic | I demonstrate flexibility in responding to tough issues |  |  |  | |  | |
| 22 | Mind Mapping | I build consensus among team members on the direction of future plans for team members |  |  |  | |  | |
| 23 | Supportive | I consistently build on others’ ideas in problem-solving discussions |  |  |  | |  | |
| 24 | Discipline | I step in and redirect destructive conflict |  |  |  | |  | |
| 25 | Empowerment | I promote recognition of team members’ contribution |  |  |  | |  | |
